# Anti-Tumor Effects of Statins in Pancreatic Ductal Adenocarcinoma Cells

**DOI:** 10.3390/ijms27072972

**Published:** 2026-03-25

**Authors:** Veronika Kucháriková, Zuzana Hatoková, Eva Baranovičová, Bibiána Baďurová, Tereza Pavlišová, Lucia Kotúľová, Michal Kalman, Juraj Marcinek, Oľga Chodelková, Slavomíra Nováková, Ján Strnádel, Henrieta Škovierová, Erika Halašová

**Affiliations:** 1 Biomedical Centre Martin, Jessenius Faculty of Medicine in Martin, Comenius University in Bratislava, Malá Hora 4C, 036 01 Martin, Slovakia; kucharikova14@uniba.sk (V.K.); zuzana.hatokova@uniba.sk (Z.H.); eva.baranovicova@uniba.sk (E.B.); bibiana.badurova@uniba.sk (B.B.); tereza.pavlisova@uniba.sk (T.P.); lucia.kotulova@uniba.sk (L.K.); ticha24@uniba.sk (O.C.); slavomira.novakova@uniba.sk (S.N.); jan.strnadel@uniba.sk (J.S.); erika.halasova@uniba.sk (E.H.); 2 Department of Medical Biochemistry, Jessenius Faculty of Medicine in Martin, Comenius University in Bratislava, Malá Hora 4D, 036 01 Martin, Slovakia; 3 Department of Pathological Anatomy, Jessenius Faculty of Medicine in Martin, Comenius University in Bratislava and University Hospital Martin, Kollárová 2, 036 01 Martin, Slovakia; michal.kalman@uniba.sk (M.K.); juraj.marcinek@uniba.sk (J.M.)

**Keywords:** pancreatic ductal adenocarcinoma, statins, personalized therapy

## Abstract

Pancreatic ductal adenocarcinoma (PDAC) has limited effective therapeutic strategies. Statins inhibit 3-hydroxy-3-methylglutaryl-coenzyme A (HMG-CoA) reductase and may affect tumor cell fitness via the mevalonate pathway, mitochondrial function, and redox homeostasis. We systematically compared seven statins in patient-derived PDAC cell lines and related viability effects to mitochondrial, redox, cell-cycle, apoptotic, and metabolic responses. Statins were tested in three PDAC cell lines (PDAC-1/2/3) using MTT assays (5–20 µM; 24–120 h). Based on MTT responses, mechanistic profiling was performed after 72 h at 20 µM concentration using lipophilic statins, including apoptosis (Annexin V/7-AAD), cell-cycle distribution, mitochondrial membrane potential (Δψm), intracellular ROS, and ^1^H-NMR quantification of intracellular and extracellular metabolites. Statins reduced viability in a concentration- and time-dependent manner, with lipophilic statins more active than hydrophilic. PDAC-1 was highly sensitive, PDAC-3 intermediate, and PDAC-2 comparatively resistant. PDAC-1 and PDAC-3 showed G0/G1 accumulation, Δψm depolarization, reactive oxygen species (ROS) elevation, and Annexin V–positive apoptosis, whereas PDAC-2 (high basal ROS) showed ROS reduction and limited apoptosis despite Δψm loss. Metabolomics indicated reduced glucose and amino-acid utilization and lactate secretion while preserving line-specific metabolic fingerprints. PDAC cell lines display marked inter-tumoral heterogeneity in statin responses, supporting evaluation of statins as chemosensitizing adjuvants in functionally guided PDAC treatment strategies.

## 1. Introduction

PDAC is one of the most lethal solid malignancies, with global five-year survival remaining below 10%. The majority of patients are diagnosed with locally advanced or metastatic disease that is inherently resistant to systemic therapy. Despite modern regimens such as FOLFIRINOX or gemcitabine-based combinations, overall survival remains limited, as PDAC is intrinsically difficult to treat and rapidly develops resistance to systemic therapy [1,2]. These limitations underscore the need for innovative therapeutic strategies, including the rational repurposing of well-characterized drugs with established safety profiles.

Statins, competitive inhibitors of HMG-CoA reductase, are widely prescribed for hypercholesterolemia, and their primary pharmacological effect is inhibition of the mevalonate pathway, thereby reducing the production of isoprenoid intermediates required for protein prenylation (farnesylation and geranylgeranylation) [3,4]. Because prenylation is essential for membrane localization and activity of small GTPases (including RAS family members) and other oncogenic signaling proteins, inhibition of the mevalonate pathway is mechanistically reasonable as an anti-tumor strategy, particularly in KRAS-driven tumors such as PDAC [1,4,5].

Beyond lipid lowering, statins exert pleiotropic effects that affect redox and inflammatory signaling, including ROS balance, nitric oxide bioavailability, and NF-κB driven cytokine programs [6]. These pleiotropic, non-lipid effects provide a biologically plausible basis for anticancer activity, particularly in KRAS-driven tumors such as PDAC, and clinical studies suggest that statins may provide benefit in oncology therapy [3,4,7].

Statins are structurally divided into natural (e.g., lovastatin), semi-synthetic (e.g., pravastatin, simvastatin), and fully synthetic agents (e.g., atorvastatin, fluvastatin, pitavastatin, rosuvastatin), which share a common dihydroxyheptanoic acid group but differ in the structure of their rings and side chains [8,9]. These structural differences determine physicochemical properties such as lipophilicity, membrane permeability, and tissue distribution. Lipophilic statins cross biological membranes more readily and accumulate to higher levels in extrahepatic tissues, which is particularly relevant for potential anticancer effects [10].

Epidemiological and clinical evidence regarding the role of statins in oncology, including pancreatic cancer, remains inconsistent, with some studies reporting reduced incidence or improved survival and others finding no clear benefit, which has fueled ongoing controversy about their true therapeutic value [2,11]. Several meta-analyses and population-based studies suggest that statin use may be associated with a reduced incidence of PDAC or improved survival, most consistently in resected patient populations, although substantial heterogeneity and confounding factors limit definitive conclusions [11,12,13]. Experimental data indicate that individual statins can exert distinct anticancer or cancer-preventive effects, especially in inflammation-driven settings, potentially involving cytokine and microenvironmental modulation [2,7,11,12,14]. In parallel, an increasing volume of preclinical evidence indicates that statins can enhance the efficacy of established chemo- and radiotherapy regimens by increasing apoptotic responses, radiosensitivity, or chemosensitivity, supporting their use primarily as chemosensitizing adjuvants rather than stand-alone cytotoxic agents [13,15,16,17].

Preclinical studies provide stronger mechanistic support for statin anticancer activity in PDAC. Several statins, particularly lipophilic compounds, have been shown to inhibit proliferation and clonogenic growth and to induce mitochondrial dysfunction in pancreatic cancer cell lines [1,15,18]. Other studies implicate cell-cycle arrest, activation of intrinsic apoptosis, suppression of YAP/TAZ and PI3K/AKT signaling, and disruption of protein prenylation as key contributors to the anti-tumor effects of statins [3,4,19]. Several of these studies highlight greater antiproliferative potency of lipophilic statins compared with hydrophilic compounds, consistent with differences in membrane permeability and intracellular accumulation [6,10,20].

Most in vitro studies have evaluated only one or a few statins at a time, typically simvastatin and/or lovastatin, in a limited number of established pancreatic cancer cell lines, which may not fully capture inter-patient heterogeneity [1,4,18,21]. The relative potency and mechanistic profiles across the full panel of clinically used statins (atorvastatin, fluvastatin, lovastatin, pitavastatin, pravastatin, rosuvastatin, simvastatin) have not been systematically compared under identical experimental conditions in three newly generated PDAC cell lines. Emerging evidence indicates that statins can exert both pro- and anti-tumor effects depending on dose, lipophilicity, cellular context, and adaptive responses such as autophagy or mitophagy, underscoring the need to integrate functional readouts of mitochondrial integrity and oxidative stress [3,6,7,13].

This study represents a comprehensive comparative evaluation of all clinically used statins in three novel, patient-derived PDAC cell lines (PDAC-1, PDAC-2, PDAC-3). Therefore, we systematically compared the effects of seven clinically available statins under identical experimental conditions in three patient-derived PDAC cell lines. First, we performed a dose- and time-dependent viability screen using the MTT assay and documented treatment-induced morphological changes by light microscopy. Based on these screening data, a standardized exposure condition was selected for downstream analyses of apoptosis, cell-cycle distribution, mitochondrial membrane potential, and ROS, and for NMR-based profiling of intracellular and extracellular metabolites. This study evaluates the potential of statins to act as chemosensitizing adjuvants that enhance tumor cell susceptibility to established chemo- or radiotherapy regimens and provides a comparative framework to define statin-specific activity patterns, relate phenotypic responses to mitochondrial and redox pathways, and identify candidates and metabolic signatures for future translational studies in PDAC.

To the best of our knowledge, this study provides a systematic analysis of statin responses across patient-derived PDAC cell lines and reveals pronounced inter-tumoral heterogeneity in mitochondrial, redox, and metabolic responses, underscoring the complexity of precision therapy development.

## 2. Results

### 2.1. Morphological Effects of Statins in Patient-Derived PDAC Cell Lines

**Figure 1:**
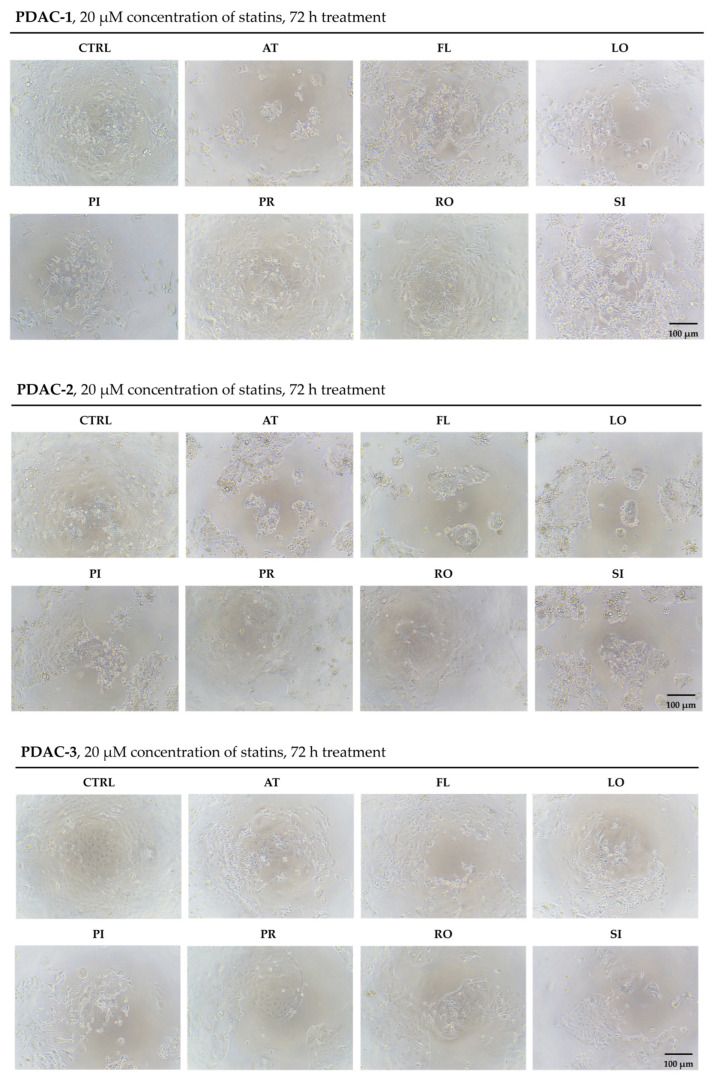
Statin-induced morphological changes in patient-derived PDAC cell lines. Representative phase-contrast images of PDAC-1, PDAC-2, and PDAC-3 cells after 72 h exposure to 20 μM concentration of statins compared with untreated control (CTRL). Each panel shows cells treated with atorvastatin (AT), fluvastatin (FL), lovastatin (LO), pitavastatin (PI), pravastatin (PR), rosuvastatin (RO), or simvastatin (SI). Images were acquired using an inverted light microscope at 10× magnification, scale bar = 100 μm. Images are representative of at least three independent biological experiments (*n* = 3).

To analyze statin-induced changes in cell morphology and organization, all three PDAC cell lines were examined by light microscopy and compared with untreated controls (Figure 1). In untreated controls, all three cell lines formed dense adherent monolayers with a relatively uniform epithelial morphology and clear cell–cell contacts. After statins treatment, all three cell lines showed visible changes in cell shape, density, and organization. In PDAC-1, the cell layer was markedly less confluent; many cells were smaller and rounded, with reduced cytoplasmic volume and partial detachment from the surface, and the continuous monolayer was replaced by small groups of remaining adherent cells surrounded by cellular debris. In PDAC-2, control cultures displayed a less compact monolayer. Statins exposure led to fragmentation of the cell layer into multiple compact aggregates separated by cell-free areas. Cells within these aggregates were often rounded and bright, and individual floating cells and debris were present in the background. In PDAC-3, statins caused a moderate decrease in cell density and disruption of the regular epithelial layer, with cells assuming more elongated or irregular shapes and forming loose networks or small clusters. Cell detachment of PDAC-3 was less extensive than in PDAC-1, but the morphology clearly differed from control cultures. Light microscopy showed that statins induced changes in confluence, cell shape and monolayer structure in all three PDAC cell lines. These qualitative observations were consistent with the strong reduction in MTT signal in PDAC-1 and PDAC-3, whereas in PDAC-2, similar stress-related morphological features occurred without a comparable decrease in MTT-derived viability.

### 2.2. Effects of Statins on Cell Viability in Patient-Derived PDAC Cell Lines

**Figure 2:**
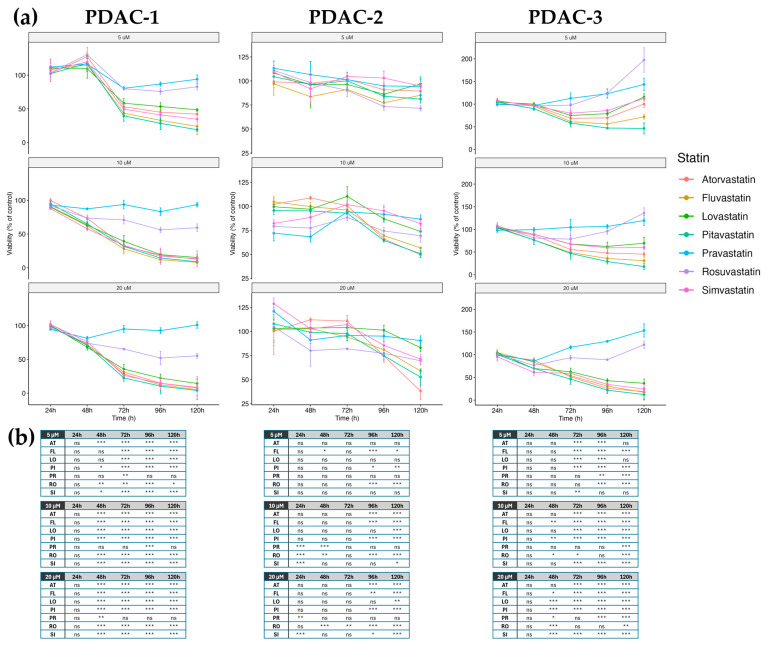
Time- and dose-dependent effects of statins on viability of patient-derived PDAC cell lines. (**a**) Cell viability was assessed by MTT assay in three PDAC cell lines (PDAC-1, PDAC-2, and PDAC-3) after treatment with seven clinically used statins: atorvastatin (AT), fluvastatin (FL), lovastatin (LO), pitavastatin (PI), pravastatin (PR), rosuvastatin (RO), and simvastatin (SI) at 5, 10, or 20 μM concentration for 24–120 h. Line graphs show viability expressed as % of DMSO-treated control for each time point; columns correspond to individual cell lines and rows to statin concentrations, with individual statins distinguished by color according to the legend. Data are presented as mean ± SD of biological triplicates (three wells per condition) (n = 3). (**b**) Summary of the statistical analysis of MTT data. A linear mixed-effects model with replicate as a random factor was used to compare each statin condition with the corresponding DMSO control; tables report estimated statin/control ratios together with significance levels coded as *** *p* < 0.001, ** *p* < 0.01, * *p* < 0.05, and ns (not significant). Numerical adjusted *p*-values for all comparisons are provided in Supplementary Figure S5.

In PDAC-1, lipophilic statins induced a marked, time- and concentration-dependent reduction in cell viability (Figure 2a). At 10 μM concentration, atorvastatin, fluvastatin, lovastatin, pitavastatin, and simvastatin significantly decreased the MTT signal at 120 h (*p* < 0.001 vs. control), whereas rosuvastatin caused a smaller but still significant reduction, and pravastatin did not differ significantly from control. At a concentration of 20 μM, all five lipophilic statins again significantly suppressed viability at 120 h. Fluvastatin and lovastatin showed the strongest inhibition, simvastatin had a slightly less pronounced effect, and atorvastatin and pitavastatin showed intermediate activity. Rosuvastatin caused only partial inhibition, and pravastatin remained without a significant effect. At earlier time points and lower concentrations, statistically significant changes were mainly observed for the most active lipophilic statins. Based on the time-course profiles in Figure 2a, PDAC-2 showed a less pronounced reduction in viability under statin treatment. At 5 μM concentration, none of the statins produced a significant change in MTT signal across the 120 h time course. At 10 μM concentration, significant differences were detected predominantly at 120 h. Atorvastatin, fluvastatin, and pitavastatin significantly reduced viability; lovastatin and rosuvastatin induced a more moderate but still significant decrease; simvastatin had a weaker effect; and pravastatin did not reach statistical significance. At 20 μM concentration, fluvastatin and pitavastatin were the most effective compounds at 120 h, followed by atorvastatin, rosuvastatin, and simvastatin, all with statistically significant reductions in viability, whereas lovastatin showed only a modest inhibitory effect and pravastatin again remained non-significant. An intermediate response profile was observed in PDAC-3. At 5 μM concentration, several lipophilic statins (atorvastatin, fluvastatin, lovastatin, pitavastatin, and simvastatin) significantly reduced viability at 72 h, while pravastatin and rosuvastatin had no significant effect. At 10 μM concentration, all five lipophilic statins significantly decreased the MTT signal at 72 and 120 h. In contrast, pravastatin and rosuvastatin did not reduce viability; at 120 h, both hydrophilic statins significantly increased the MTT readout compared with control. At 20 μM concentration, the inhibitory pattern for lipophilic statins was maintained at 72 and 120 h, whereas pravastatin and, to a lesser extent, rosuvastatin were associated with higher MTT values than controls at 120 h. Detailed estimates of statin/control ratios and corresponding *p*-values for all conditions (cell line, concentration, time and statin) are provided in Figure 2b.

### 2.3. Statins Differentially Modulate Intracellular ROS Levels in PDAC Cells

**Figure 3:**
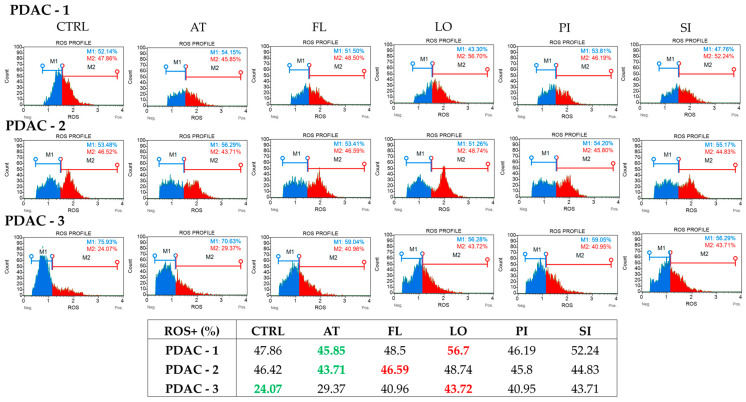
Intracellular (ROS) profiles in patient-derived PDAC cell lines after statin treatment. ROS levels were measured with the Muse™ Oxidative Stress Assay after 72 h exposure to 20 μM concentration of atorvastatin (AT), fluvastatin (FL), lovastatin (LO), pitavastatin (PI), or simvastatin (SI) in three patient-derived PDAC cell lines. For PDAC-1, PDAC-2, and PDAC-3 (rows), representative histograms are shown for untreated control cells (CTRL) and for each statin (columns); blue peaks (M1) correspond to ROS-negative cells and red peaks (M2) to ROS-positive cells. The table below summarizes the proportion of ROS-positive cells (ROS+ %) for every treatment condition in the three PDAC lines. Within each line, values highlighted in green denote the lowest ROS+ fraction (greatest ROS reduction), whereas values in red denote the highest ROS+ fraction (greatest ROS increase). Additional statistical visualization is shown in Supplementary Figure S1.

Intracellular ROS levels were analyzed using the Muse™ Oxidative Stress Kit after 72 h exposure to 20 μM concentration of lipophilic statins (atorvastatin, fluvastatin, lovastatin, pitavastatin, and simvastatin) to investigate whether statins treatment alters redox homeostasis in PDAC cells (Figure 3). Histograms illustrate the distribution of ROS-negative (M1) and ROS-positive (M2) populations for each treatment and cell line are shown in Figure 3, together with the percentage of ROS-positive cells. In PDAC-1 cells, the basal proportion of ROS-positive cells in untreated controls was approximately 48%. Atorvastatin slightly decreased the ROS-positive fraction (46%), whereas fluvastatin and pitavastatin had only minor effects, remaining close to control values (49% and 46%, respectively). In contrast, lovastatin and simvastatin increased the ROS-positive population to 57% and 52%, respectively, indicating a shift toward a more oxidizing intracellular environment in response to these two statins. In PDAC-2, control cells displayed the highest basal oxidative status among the three cell lines, with 55% ROS-positive cells. All tested statins reduced the ROS-positive fraction, with values ranging from 44–49%. Atorvastatin showed the strongest decrease (44%), followed by pitavastatin, fluvastatin, simvastatin, and lovastatin. In PDAC-2 cells, statins tended to attenuate, rather than enhance, ROS levels. In PDAC-3, the untreated control exhibited the lowest basal ROS burden (24% ROS-positive cells). In this context, all five statins increased ROS production, with atorvastatin raising the ROS-positive fraction to 29%, and fluvastatin, pitavastatin, simvastatin, and especially lovastatin elevating it to 41–44%. The most pronounced shift was observed for lovastatin (44% ROS-positive cells), indicating a robust pro-oxidant effect in this cell line.

### 2.4. Statin-Induced Alterations in Mitochondrial Membrane Potential in PDAC Cells

**Figure 4:**
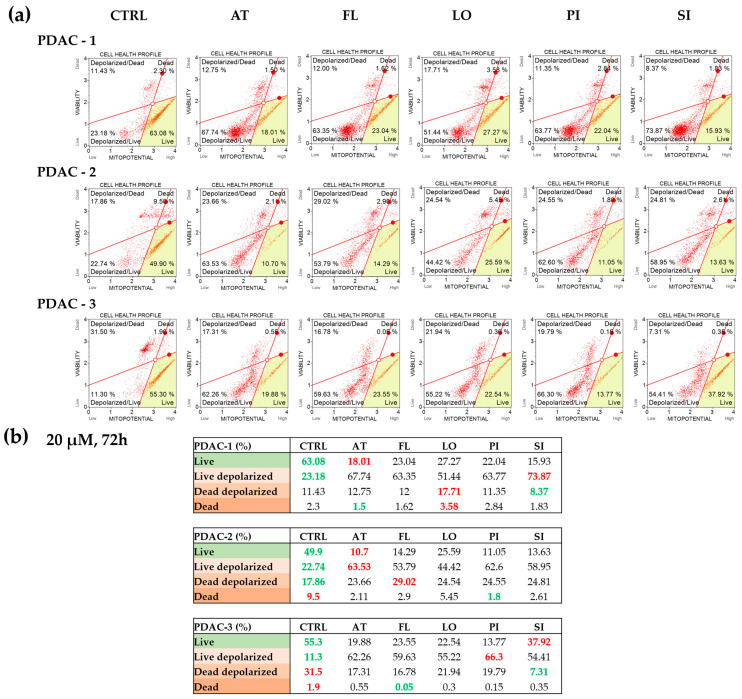
Effects of statins on mitochondrial membrane potential in patient-derived PDAC cell lines. (**a**) Mitochondrial membrane potential was assessed using the Muse™ MitoPotential Assay after 72 h exposure to 20 μM concentration of atorvastatin (AT), fluvastatin (FL), lovastatin (LO), pitavastatin (PI), or simvastatin (SI) in three patient-derived PDAC cell lines. For PDAC-1, PDAC-2, and PDAC-3 (rows), representative dot plots are shown for untreated control cells (CTRL) and for each statin (columns). Each plot is divided into four populations: live cells with preserved mitochondrial membrane potential (Live), live cells with depolarized mitochondria (Live depolarized), depolarized but still viable cells (Dead depolarized), and dead cells (Dead). (**b**) The corresponding tables summarize the percentage of cells in each population for all treatment conditions in the three PDAC lines. Within each line, values highlighted in green indicate the most favorable pattern (highest Live fraction and/or lowest depolarized/dead fractions), whereas values highlighted in red indicate the least favorable pattern (lowest Live fraction and/or highest depolarized/dead fractions). Additional statistical visualization is shown in Supplementary Figure S2.

Mitochondrial membrane potential was evaluated using the Muse™ MitoPotential assay after 72 h exposure to 20 μM concentration of lipophilic statins in all three PDAC cell lines (Figure 4). In untreated controls, most cells in each line were classified as live with intact Δψm, with smaller fractions of live depolarized, depolarized/dead, and dead cells. Statins treatment in all cell lines led to a marked decrease in the proportion of live cells with preserved Δψm and a concomitant increase in live depolarized cells, indicating mitochondrial depolarization rather than immediate loss of viability. Atorvastatin, pitavastatin, and simvastatin produced the most pronounced shift toward depolarized populations in PDAC-1 and PDAC-2, while fluvastatin and lovastatin also increased depolarized/dead fractions, particularly in PDAC-2. In PDAC-3, all statins increased the proportion of depolarized live cells to more than 50%, while the fraction of dead cells remained very low, indicating marked mitochondrial dysfunction with only limited cell death under these conditions. These data show that lipophilic statins consistently impair mitochondrial membrane potential in PDAC cells in a statin- and cell line-dependent manner.

### 2.5. Effects of Statins on Cell-Cycle Distribution in PDAC Cells

**Figure 5:**
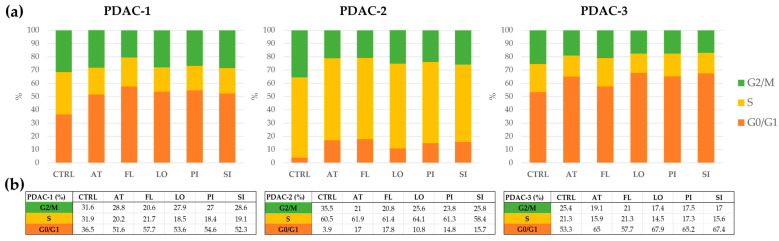
Cell-cycle distribution after statins treatment in patient-derived PDAC cell lines. (**a**) PDAC-1, PDAC-2, and PDAC-3 cells were exposed to 20 μM concentration of atorvastatin (AT), fluvastatin (FL), lovastatin (LO), pitavastatin (PI), or simvastatin (SI) for 72 h, and DNA content was analyzed using the Muse™ Cell Cycle Assay. Segmented bar graphs show, for each cell line (separate panels) and each condition (CTRL, AT, FL, LO, PI, SI), the percentage of cells in G0/G1, S, and G2/M phases, as indicated in the legend (orange, yellow, and green, respectively). (**b**) The table reports the corresponding numerical percentages for each cell-cycle phase to facilitate direct comparison across cell lines and conditions, including relative shifts compared with the corresponding control within each cell line. Additional statistical visualization is shown in Supplementary Figure S3.

Across all three PDAC cell lines, exposure to lipophilic statins produced characteristic but clearly distinct alterations in cell-cycle distribution (Figure 5). In PDAC-1, treatment with atorvastatin, fluvastatin, lovastatin, pitavastatin, and simvastatin shifted the population from a more evenly distributed profile in untreated controls toward a pattern dominated by G0/G1 phase, accompanied by a visible reduction in S-phase and a modest decline in G2/M phase. This indicates an efficient G1/S blockade and a pronounced cytostatic effect. PDAC-3, which already exhibited a predominance of G0/G1 cells at baseline, showed a further enrichment of the G0/G1 fraction with a parallel decrease in S-phase after statin treatment, while G2/M remained relatively stable, consistent with additional slowing of cell-cycle progression at the G1 checkpoint. By contrast, PDAC-2 maintained a highly proliferative phenotype: control cells were largely confined to S and G2/M phases with only a small G0/G1 compartment, and statin exposure induced only minor increases in G0/G1 and slight reductions in S-phase. Overall, the cell-cycle profiles demonstrate that lipophilic statins induce a robust G0/G1 accumulation in PDAC-1 and PDAC-3, whereas PDAC-2 displays only limited sensitivity to statin-induced cell-cycle arrest, underscoring substantial inter-tumoral variability in the cytostatic response.

### 2.6. Pro-Apoptotic Effects of Lipophilic Statins in Patient-Derived PDAC Cell Lines

**Figure 6:**
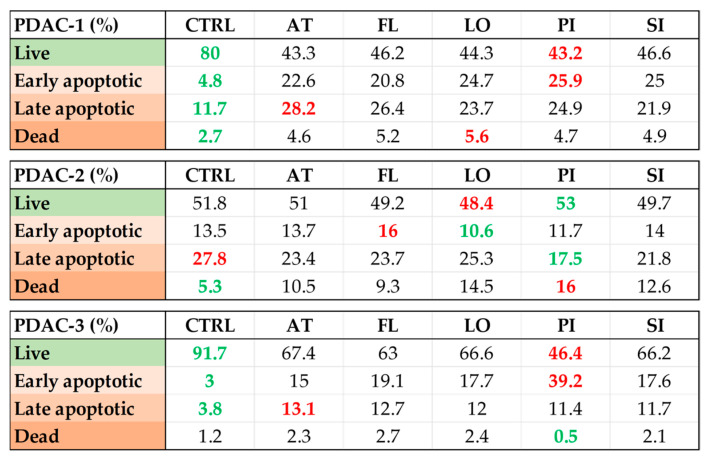
Pro-apoptotic effects of lipophilic statins in patient-derived PDAC cell lines. Apoptosis was quantified by Annexin V/7-AAD staining after 72 h exposure to 20 μM concentration of atorvastatin (AT), fluvastatin (FL), lovastatin (LO), pitavastatin (PI), or simvastatin (SI) in three patient-derived PDAC cell lines (PDAC-1, PDAC-2, and PDAC-3). For each line, the table reports the percentages of live, early apoptotic, late apoptotic, and dead cells under control conditions (CTRL) and after treatment with each statin. Values highlighted in green indicate the most favorable pattern within a given cell line (highest proportion of live cells and/or lowest apoptotic and dead fractions), whereas values highlighted in red indicate the most pro-apoptotic pattern (lowest live fraction and/or highest late apoptotic or dead fractions). Additional statistical visualization is shown in Supplementary Figure S4.

Apoptosis was quantified by Annexin V-APC/7-AAD staining and flow cytometry after 72 h exposure to 20 μM concentration of lipophilic statins in all three PDAC cell lines (Figure 6). In PDAC-1, control cultures contained 80% live cells and 17% Annexin V-positive cells, whereas all statins markedly reduced the live fraction to 43–47% and increased both early (21–26%) and late (22–28%) apoptotic cells, while the proportion of dead cells remained relatively low (approximately 4–6%), indicating a strong shift toward apoptosis. Basal apoptosis was intermediate (41% Annexin V-positive cells in controls) and statin treatment produced only moderate changes in PDAC-2, with the live population remaining around 48–53%, early apoptosis at 11–16%, and late apoptosis slightly decreased or comparable to control, although pitavastatin and lovastatin increased the dead fraction to 14–16%. In PDAC-3, controls were predominantly viable (92% live, approximately 7% Annexin V-positive), and all statins reduced the live fraction (to 46–67%) and increased early and late apoptotic populations; the most substantial effect was observed with pitavastatin, which lowered viable cells to 46% and raised early apoptosis to 39%, whereas late apoptotic and dead fractions remained low. These data indicate that lipophilic statins induce apoptosis in a cell line-dependent manner, with PDAC-1 and PDAC-3 showing a robust apoptotic response compared to PDAC-2 exhibiting relative resistance under the same conditions.

### 2.7. Statins-Induced Alterations in Intra- and Extracellular Metabolism of PDAC Cells

**Figure 7:**
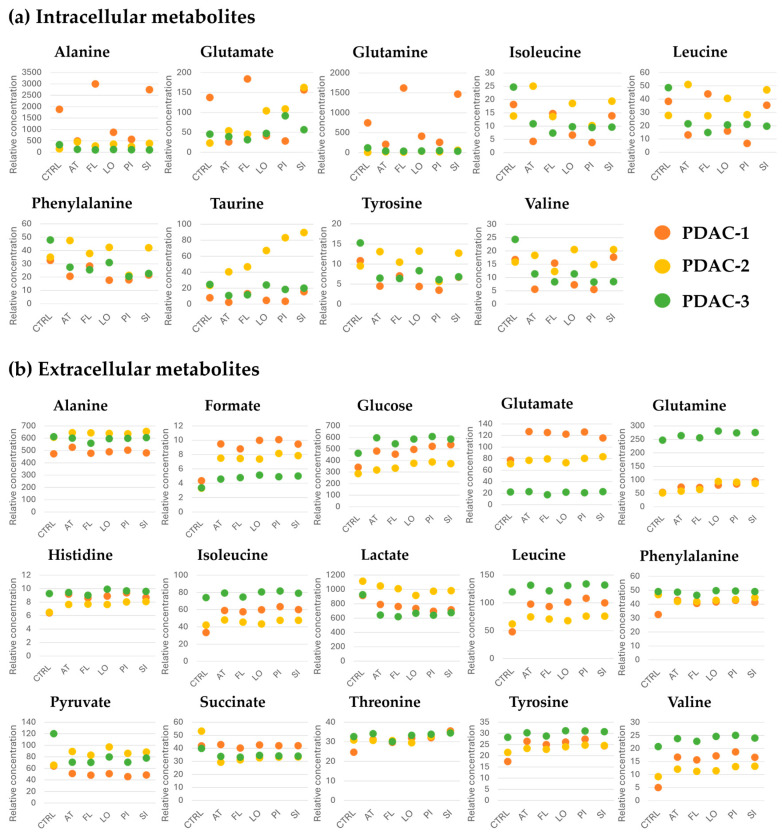
Statins-induced metabolic alterations in patient-derived PDAC cell lines. Three patient-derived PDAC cell lines (PDAC-1, PDAC-2, and PDAC-3) were treated for 72 h with 20 μM concentration of atorvastatin (AT), fluvastatin (FL), lovastatin (LO), pitavastatin (PI), or simvastatin (SI), or left untreated (CTRL), and metabolite levels were quantified by ^1^H-NMR spectroscopy. (**a**) Dot plots of intracellular alanine, glutamate, glutamine, isoleucine, leucine, phenylalanine, taurine, tyrosine, and valine, whereas panel, (**b**) Dot plots of extracellular alanine, formate, glucose, glutamate, glutamine, histidine, isoleucine, lactate, leucine, phenylalanine, pyruvate, succinate, threonine, tyrosine, and valine measured in the corresponding culture media. In both panels, each point represents the relative concentration of the indicated metabolite under a given treatment condition (CTRL, AT, FL, LO, PI, SI); PDAC-1, PDAC-2, and PDAC-3 are distinguished by color according to the legend.

Analysis of culture media revealed pronounced metabolic differences among the PDAC cell lines and their response to statins treatment (Figure 7). Under control conditions, PDAC-2 cells exhibited the highest glucose consumption and lactate production, whereas PDAC-3 showed the lowest glycolytic flux. Statins treatment reduced glucose uptake and lactate release in all three cell lines; however, PDAC-2 consistently maintained higher values compared with PDAC-1 and PDAC-3. Depletion of branched-chain amino acids (leucine, isoleucine, and valine) in the culture media followed a similar pattern to glucose consumption, with the greatest uptake observed in PDAC-2 and the lowest in PDAC-3. Intracellular analysis showed elevated BCAA levels in untreated PDAC-3 cells, consistent with limited BCAA utilization, while statin-treated PDAC-2 cells displayed increased intracellular BCAA accumulation compared with their untreated counterparts. Statin treatments also differentially affected pyruvate levels across the cell lines. Extracellular pyruvate decreased in PDAC-1 and PDAC-3 following statins exposure, whereas PDAC-2 cells showed increased pyruvate accumulation in the media. Distinct patterns were observed for glutamine and glutamate: PDAC-3 cultures exhibited higher extracellular glutamine levels and lower glutamate concentrations compared with PDAC-1 and PDAC-2. Phenylalanine depletion was most pronounced in PDAC-1 under control conditions, and PDAC-3 maintained the highest levels of both phenylalanine and tyrosine in culture media regardless of statin treatment. In addition, PDAC-1 cells displayed low extracellular pyruvate and alanine levels together with marked accumulation of alanine in cell lysates, a phenotype that was further enhanced by statin treatment. This intracellular alanine enrichment was not observed in PDAC-2 or PDAC-3 cells.

## 3. Discussion

Statins are increasingly recognized as modulators of tumor cell survival, and preclinical studies have shown dose- and time-dependent reductions in viability across solid tumors, including PDAC [3,4,22]. In established PDAC cell lines, simvastatin, lovastatin, and atorvastatin inhibit proliferation and clonogenicity and promote cell-cycle arrest and apoptosis [15,18,19]. Using three newly established patient-derived PDAC cell lines under strictly standardized conditions, our data extend this evidence by showing that both the magnitude and kinetics of the antiproliferative response depend on the specific statin and the tumor background.

Across all cell lines, we observed a clear and reproducible potency pattern: lipophilic statins (atorvastatin, fluvastatin, lovastatin, pitavastatin, and simvastatin) were consistently more effective than the hydrophilic statins (pravastatin and rosuvastatin). This aligns with known differences in membrane permeability and tissue distribution, where lipophilic statins accumulate more readily in extrahepatic cells and thus can exert stronger anti-tumor effects [6,10,20]. In line with prior reports in pancreatic and other carcinoma models, pravastatin was largely ineffective at comparable concentrations, whereas lovastatin and simvastatin produced pronounced functional effects [15,23,24]. Fluvastatin and pitavastatin also emerged among the more active lipophilic compounds, supporting the importance of direct head-to-head comparisons across statins.

A key finding is the marked inter-tumoral heterogeneity captured by the three patient-derived lines. PDAC-1 was highly sensitive, showing robust dose- and time-dependent reductions in viability with all lipophilic statins. PDAC-2 was comparatively resistant, requiring higher concentrations and longer exposures for significant effects, with incomplete inhibition even under the most intensive conditions. PDAC-3 showed an intermediate response to lipophilic statins but a distinct behavior with hydrophilic statins at later time points, where MTT values were preserved or slightly increased. Because the MTT assay reports cellular reductase activity rather than directly quantifying cell abundance, this pattern most likely reflects altered metabolic activity in surviving cells rather than true growth stimulation, especially given the mechanistic effects of lipophilic statins observed in this line (Figure 2) [25,26].

Our multi-parameter profiling indicates that statins induce a common upstream stress signal, but the downstream outcome is determined by the cellular context. ROS are central regulators of tumor cell behavior, and statin effects on ROS are known to be context-dependent, varying from pro-oxidant responses in tumor cells to antioxidant effects in cardiovascular tissues via modulation of NADPH oxidases and antioxidant programs such as Nrf2/HO-1 [27,28,29,30,31,32,33,34]. In our cell lines, PDAC-1 and PDAC-3, which had low to intermediate basal ROS, tended to respond to lipophilic statins with ROS elevation (most prominently with lovastatin and simvastatin). PDAC-2, characterized by the highest basal ROS burden, consistently showed ROS attenuation. Notably, ROS elevation co-occurred with stronger cytostatic and apoptotic phenotypes (PDAC-1 and PDAC-3), whereas ROS attenuation aligned with a more adaptive, resistant phenotype (PDAC-2). It suggests that baseline redox state and the direction of statin-induced ROS change shape whether statin pressure promotes cytotoxic programs or supports adaptive stress responses.

Mitochondrial dysfunction was another consistent feature. Statins can impair mitochondrial respiration through multiple mechanisms, including reduced coenzyme Q10 availability and electron transport chain interference, resulting in mitochondrial membrane potential (Δψm) depolarization and activation of intrinsic apoptosis pathways [34,35,36]. In our patient-derived PDAC cell lines, lipophilic statins induced pronounced Δψm depolarization in all three cell lines, indicating that mitochondrial dysfunction is a shared upstream effect. However, Δψm loss alone did not predict cytotoxicity: PDAC-2 displayed substantial depolarization without a comparably strong cytostatic/apoptotic phenotype, consistent with the idea that mitochondrial stress can remain sublethal when downstream apoptotic is not activated (e.g., by survival signaling or protective autophagy/mitophagy programs) [7,13,37,38]. In PDAC-1 and PDAC-3, Δψm depolarization coincided with ROS elevation and stronger apoptotic responses, supporting functional coupling between oxidative stress and mitochondrial collapse described in mechanistic studies [15,24]. In contrast, PDAC-2 maintained ROS attenuation despite Δψm loss, suggesting that in this line mitochondrial effects may be driven more directly by respiratory interference than by ROS amplification.

Cell-cycle and apoptosis readouts clearly separated sensitive and resistant phenotypes. Statins frequently induce G1/S arrest through mevalonate pathway inhibition and impaired prenylation of small GTPases, with downstream changes in cyclins/CDKs and CDK inhibitors [3,4,13,22]. In our study, PDAC-1 and PDAC-3 showed robust G0/G1 accumulation with reduced S-phase, indicating effective cytostatic arrest. PDAC-2 exhibited a hyperproliferative baseline profile dominated by S+G2/M and showed only modest redistribution after statins exposure, consistent with reduced sensitivity of checkpoint control to statin-mediated pathway inhibition. While the observed changes in cell cycle distribution indicate a statin-associated redistribution under our experimental conditions, the present study was designed as a functional phenotypic profiling across statins and PDAC cell lines and was not intended to comprehensively define the underlying regulators. Further work will extend these phenotypic observations by applying a dedicated quantitative metric of arrest magnitude across cell lines, for example based on control-normalized shifts in G0/G1 and S-phase fractions, and by performing mechanistic validation through assessment of key cell cycle regulators such as p21 and Cyclin D1, together with related checkpoints and upstream signaling pathways. Apoptosis measurements aligned with this stratification: PDAC-1 and PDAC-3 displayed clear induction of Annexin V–positive apoptosis across lipophilic statins (with particularly strong effects in PDAC-3 after pitavastatin), whereas PDAC-2 showed comparatively modest shifts in apoptotic compartments under identical conditions despite mitochondrial depolarization. Together, these data indicate that statin responsiveness is defined by the integrated capacity to couple mitochondrial/redox stress to checkpoint enforcement and apoptotic commitment rather than by any single mechanistic event.

Comparative analysis of untreated cells revealed that PDAC-2 consumed substantially more glucose and produced higher levels of lactate than PDAC-1 and PDAC-3, indicating enhanced glycolytic activity and a stronger dependence on the Warburg effect [39]. Although statins treatment led to an overall reduction in glucose consumption and lactate production relative to untreated conditions in all three cell lines, PDAC-2 consistently maintained higher glucose uptake and lactate release than both PDAC-1 and PDAC-3, even in the presence of statins. The lowest glucose consumption and lactate production observed in the PDAC-3 line for both untreated and treated cells suggest that its overall glycolytic activity is reduced and likely reflects intrinsic metabolic characteristics specific to this cell line. The parallel depletion of glucose and branched-chain amino acids (BCAAs: leucine, isoleucine, and valine) observed across all three cell lines suggests a tight coupling between energy metabolism and amino acid utilization. Cells with higher glycolytic activity, namely PDAC-1 and PDAC-2, not only consumed more glucose but also took up more BCAAs, which likely reflects increased anabolic demands for protein synthesis and anaplerotic replenishment of the TCA cycle. Under control conditions, these two cell lines also showed lower intracellular levels of BCAAs in cell lysates compared with PDAC-3, further supporting their enhanced capacity for amino acid uptake and metabolic utilization. The reduced uptake of both glucose and BCAAs from the culture media upon statin treatment indicates that statins impair overall metabolic activity, potentially by limiting cell viability and biosynthetic capacity. These findings highlight the coordinated regulation of carbohydrate and amino acid metabolism in cancer cells. In cell lysates from statin-treated cells, the pattern of BCAA abundance was not entirely uniform; however, the PDAC-2 cell line exhibited the highest intracellular BCAA levels, in some cases exceeding those observed in control cells, suggesting reduced BCAA utilization compared with untreated PDAC-2. Statin treatment differentially affected the extracellular levels of pyruvate across the three PDAC cell lines. In PDAC-1 and PDAC-3, statin exposure led to decreased amounts of pyruvate in the culture media, consistent with a reduction in overall glycolytic activity. In contrast, PDAC-2 cells, which exhibited the highest glucose and BCAA consumption, showed increased pyruvate levels upon statin treatment. This observation may reflect the metabolic plasticity of PDAC-2, in which inhibition of cholesterol biosynthesis and associated cellular stress leads to altered glycolytic processing and increased pyruvate excretion rather than its immediate use in the TCA cycle or anabolic pathways. These results highlight the importance of cell line–specific metabolic programs in determining the response to statins and suggest that highly glycolytic and metabolically flexible tumor cells may adapt to metabolic stress by rerouting key metabolic intermediates such as pyruvate.

The observed differences in glutamine and glutamate dynamics across the PDAC cell lines appear to correlate with their clinical and pathological backgrounds. PDAC-1 and PDAC-2, which displayed higher glycolytic activity and enhanced glutamine consumption, were derived from primary pancreatic tumors with intermediate histological grades (PDAC-1: G3, pT3 pN1; PDAC-2: G2, pT2 pN2) in patients aged 49 and 60, respectively. These tumors likely exhibit higher proliferative activity, consistent with the preferential utilization of glutamine as an anabolic fuel for rapidly dividing cells [40]. In contrast, PDAC-3, originating from a high-grade PDAC (G3) in a 64-year-old patient who underwent total duodenopancreatectomy, showed lower glycolytic activity, elevated glutamine levels in the medium, and marked glutamate depletion. This metabolic profile suggests an alternative energy strategy in which glutamate may serve primarily as a TCA cycle substrate. Such a shift could reflect increased metabolic plasticity of more aggressive, less glycolytically active tumors that can efficiently use alternative carbon sources depending on nutrient availability and the expression of specific transporters and enzymes. Exposure to statins led to slightly increased glutamine levels in the culture media in all three cell lines, indicative of reduced glutamine utilization. PDAC-1 cells exhibited a more pronounced accumulation of extracellular glutamate following statin treatment compared with PDAC-2 and PDAC-3. No consistent pattern of statin effects was observed in the cell lysates: both glutamine and glutamate levels increased in PDAC-1 and PDAC-2 cells upon fluvastatin and simvastatin treatment; these observations are based on a single measurement and therefore do not allow definitive conclusions. A very similar pattern of metabolite levels to that seen for BCAAs was also observed for the amino acids phenylalanine, lysine, histidine, and tyrosine in the culture media, both in control cells and after statin treatment. This finding is in line with the proposed relationship between glycolytic activity and amino acid consumption. Likewise, intracellular levels of phenylalanine and tyrosine in cell lysates followed a pattern comparable to that of BCAAs, both in terms of baseline differences between cell lines and in response to statin exposure, further supporting this interpretation. Taken together, our results indicate that statin treatment broadly reduces metabolic activity in PDAC cells, as evidenced by decreased uptake of glucose and amino acids, lower lactate production, and altered glutamine–glutamate handling, but the magnitude and nature of these effects are cell line specific. Importantly, none of the individual statins produced a clearly distinct metabolic profile; instead, all lipophilic statins tended to induce qualitatively similar changes in extracellular and intracellular metabolites across the three PDAC lines.

Statins treatment induced comparable upstream stress responses in all three PDAC cell lines, including mitochondrial dysfunction and morphological alterations. PDAC-1 and PDAC-3 shared a coordinated response pattern in which statin exposure was accompanied by ROS elevation, G0/G1 cell-cycle accumulation, induction of Annexin V–positive apoptosis and attenuation of metabolic activity, resulting in a strong (PDAC-1) or intermediate (PDAC-3) reduction in viability. In contrast, PDAC-2, characterized by high basal ROS, a hyperproliferative cell-cycle profile, and high metabolic adaptability, showed ROS attenuation, minimal cell-cycle redistribution, and preserved metabolic activity under statins treatment, with no corresponding reduction in viability. Together, these findings indicate that statin responsiveness in PDAC is defined by the integrated capacity to couple redox, cell-cycle, apoptotic, and metabolic responses rather than by any single mechanistic alteration (Table 1).

Overall, our data show that the response of patient-derived PDAC cell lines to statins is formed by interactions between redox balance, cell-cycle regulation, and apoptotic capacity and varies markedly between individual tumors [3,4,7,21,22]. PDAC-1 and PDAC-3, which exhibit lower basal ROS, are more susceptible to statin-induced ROS elevation, mitochondrial depolarization, G_0_/G_1_ accumulation, and Annexin V–positive apoptosis, whereas PDAC-2, characterized by high basal ROS and a hyperproliferative cell-cycle profile, responds with ROS reduction, only modest cytostatic effects, and relative resistance to apoptosis despite loss of mitochondrial membrane potential, consistent with a stronger reliance on anti-apoptotic signaling and autophagy-based protective mechanisms [13,34,37,38]. Preclinical data across multiple tumor types suggest that statins may be more appropriately explored as chemosensitizing adjuvants than as classical cytotoxic agents. In our study, statins can modulate PDAC cell fitness and associated phenotypes, which provides a rationale to directly test statins in combination regimens in future work. Published studies report that lovastatin enhances gemcitabine efficacy in PDAC; fluvastatin increases radiosensitivity by modulating DNA damage responses; and autophagy, and atorvastatin, simvastatin, or pitavastatin augment therapy induced apoptosis in combination with platinum-based drugs or radiation [13,15,16,17,41]. The incomplete loss of viability observed in our PDAC cell lines, even under maximally effective statin conditions, is consistent with this concept and suggests that statins primarily modulate tumor cell fitness, mitochondrial function, and redox homeostasis in ways that can be exploited to improve responses to standard PDAC therapies [23,24,34]. The clearly divergent functional profiles of PDAC-1, PDAC-2, and PDAC-3 underscore marked inter-tumoral heterogeneity and suggest that different patients are likely to display distinct statin sensitivity patterns [4,21]. These findings support the implementation of personalized, functionally guided therapeutic strategies that use patient-derived PDAC cell lines and integrated viability, ROS, mitochondrial, cell-cycle, and apoptosis readouts to identify those tumor subsets most likely to benefit from statin-based combination regimens [7,19,22]. Taken together, these findings could provide a functional preclinical framework to stratify patient-derived PDAC cell lines by statin responsiveness using integrated viability, ROS, mitochondrial, cell-cycle, and apoptosis readouts. This approach should motivate further evaluation of statin-based combination strategies in appropriate in vivo PDAC models before considering clinical translation [7,19,22].

### Limitations of Study

This study uses patient-derived PDAC cell lines as in vitro systems to functionally profile statin responses under standardized conditions. These models do not capture key features of the tumor microenvironment, including immune and stromal components, fibroblast interactions, and extracellular matrix context, all of which can substantially influence therapeutic responses. Therefore, the present results cannot address how microenvironmental signaling may modulate statin-associated effects observed here. Another limitation is the limited number of PDAC cell lines included, so the findings should be confirmed in additional patient-derived models. Future work should validate these observations in more complex systems such as co-cultures or organoids and in appropriate in vivo PDAC models.

## 4. Materials and Methods

### 4.1. Cell Lines and Ethical Approval

Three PDAC cell lines (PDAC-1, PDAC-2, PDAC-3) were established in our laboratory from surgically resected tumors obtained from patients diagnosed with PDAC (Table 2) [42]. Patients (the donors of tumor tissue) signed an informed consent letter and donated part of the tissue for the project, previously approved by the Ethics Committee of the Jessenius Faculty of Medicine, Comenius University in Bratislava (approval No. EK 1946/2017).

### 4.2. Cell Culture

Cells were maintained in Dulbecco’s Modified Eagle Medium (DMEM; Gibco, Thermo Fisher Scientific, Waltham, MA, USA), supplemented with 10% fetal bovine serum (FBS; Gibco, Thermo Fisher Scientific, Waltham, MA, USA), 100 U/mL penicillin (Biosera, Cholet, France) and 100 μg/mL streptomycin (Biosera, Cholet, France). Cells were cultivated at 37 °C in a humidified atmosphere containing 5% CO_2_ and subcultured using 0.25% TrypLE™ Express (Gibco, Thermo Fisher Scientific, Waltham, MA, USA). Cells were seeded at a density of 5 × 10^3^ cells/cm^2^, and all experiments were performed between passages 4 and 7.

### 4.3. Drug Treatment

Statins used in this study included atorvastatin (PHR1422, Merck KGaA, Darmstadt, Germany), fluvastatin (PHR1620, Merck KGaA, Darmstadt, Germany), lovastatin (PHR1285, Merck KGaA, Darmstadt, Germany), pitavastatin (SML2473, Sigma–Aldrich, St. Louis, MO, USA), pravastatin (PHR3407, Merck KGaA, Darmstadt, Germany), rosuvastatin (PHR1928, Merck KGaA, Darmstadt, Germany), and simvastatin (PHR1438, Merck KGaA, Darmstadt, Germany). Stock solutions (10 mM) were prepared in 100% Dimethyl sulfoxide (DMSO; Sigma–Aldrich, St. Louis, MO, USA) and stored at −20 °C protected from light.

For the MTT cell-viability assay, cells were treated with 5, 10, or 20 μM concentration of each statin for 24, 48, 72, 96, or 120 h. Based on the concentration- and time-dependent viability responses observed in the MTT assay, 20 μM concentration of statins treatment for 72 h was selected as the standard exposure for all subsequent assays (Annexin V apoptosis, cell-cycle analysis, mitochondrial membrane potential, ROS quantification, and metabolomics). These conditions were chosen based on our previous experience and preliminary experiments in the PDAC cell lines to obtain a measurable and reproducible response while avoiding excessive cell death that could interfere with subsequent assays. The same conditions were applied to all statins to allow direct comparison. Furthermore, the selected concentration and exposure duration are consistent with commonly used in vitro conditions reported in the literature, which supports comparability across studies. In these mechanistic experiments, only lipophilic statins (atorvastatin, fluvastatin, lovastatin, pitavastatin, and simvastatin) were used because of their higher membrane permeability and more efficient intracellular uptake compared with hydrophilic statins. Untreated cells served as negative controls. The final DMSO (Sigma–Aldrich, St. Louis, MO, USA) concentration did not exceed 0.5% (*v*/*v*) under analyzed condition.

### 4.4. Cell-Viability Assay and Light Microscopy

Cell viability was measured using the MTT assay. Cells were seeded into 96-well plates (Costar, Corning Incorporated, Corning, NY, USA) at 3 × 10^3^ cells/well and allowed to adhere for 24 h. After statins exposure, the medium was replaced with fresh medium containing 10 μL 3-(4,5-dimethylthiazol-2-yl)-2,5-diphenyltetrazolium bromide (MTT) solution (5 mg/mL; Duchefa Biochemie B.V., Haarlem, The Netherlands) per well, followed by incubation for 4 h at 37 °C. Formazan crystals were dissolved overnight in 5% Sodium dodecyl sulfate (SDS; Sigma–Aldrich, St. Louis, MO, USA) at 37 °C. Absorbance was measured at 570 nm with 650 nm as a reference, using an Epoch microplate reader (BioTek, Winooski, VT, USA). Cell viability was expressed as a percentage of untreated controls. Experiments were performed in three technical triplicates. Cell morphology was also examined at each time point by light microscopy using an inverted light microscope with 10× magnification.

### 4.5. Apoptosis Assay

Apoptosis was quantified using the APC Annexin V Apoptosis Detection Kit with 7-AAD (BioLegend, San Diego, CA, USA) according to the manufacturer’s protocol. After 72 h statin exposure, both floating and adherent cells were collected for Annexin V/7-AAD analysis. Cells were detached with TrypLE™ Express (Gibco, Thermo Fisher Scientific, Waltham, MA, USA), washed twice with Dulbecco’s Phosphate-Buffered Saline (DPBS; Biosera, Cholet, France), and centrifuged at 300× *g* for 5 min at 13 °C. Pellets were resuspended in 1× Annexin V Binding Buffer (1 × 10^6^ cells/mL), and 1 × 10^5^ cells were stained with APC Annexin V and 7-AAD for 15 min at room temperature (RT) in the dark. Samples were analyzed within 1 h using a flow cytometer (FACS Aria™ Cell Sorter, BD Biosciences, San Jose, CA, USA). Analysis was performed on 10,000 events, and the gating was set up based on an unstained/isotype control, while the gating in all samples of each statin was consistent. Data were processed to determine proportions of viable, early apoptotic, late apoptotic/secondary necrotic, and necrotic cells.

### 4.6. Analysis of Cellular Processes

Analyses of cell-cycle distribution, mitochondrial membrane potential, and intracellular ROS were performed using Muse™ reagent kits in combination with the Muse™ Cell Analyzer (Luminex Corporation, Austin, TX, USA). All procedures were carried out according to the manufacturer’s instructions. After 72 h exposure to 20 μM concentration of lipophilic statins cells were then detached with TrypLE™ Express (Gibco, Thermo Fisher Scientific, Waltham, MA, USA) at 37 °C, washed once with DPBS, and centrifuged at 300× *g* for 5 min at 13 °C. Cells were counted and adjusted to the required concentration, resuspended in the appropriate assay buffers, and processed according to the specific staining protocols for each Muse™ assay.

#### 4.6.1. Cell-Cycle Analysis

First, 3 × 10^5^ cells were fixed with 70% (*v*/*v*) cold ethanol (Centralchem, Bratislava, Slovakia) and stored at −20 °C until analysis (for at least 3 h). On the day of staining, cells were washed with DPBS, resuspended in Muse™ Cell Cycle Reagent (Luminex Corporation, Austin, TX, USA), and incubated for 30 min at RT in the dark. Samples were analysed using a Guava^®^ Muse™ Cell Analyzer (Luminex Corporation, Austin, TX, USA), and the proportions of cells in the G0/G1, S, and G2/M phases were determined. The proliferation index (PI) was calculated using the following equation [43]:


PI (%) = (S + G2/M)/(G0/G1 + S + G2/M) × 100
(1)

#### 4.6.2. Mitochondrial Membrane Potential

Mitochondrial membrane potential was evaluated using the Muse™ MitoPotential Kit (Luminex Corporation, Austin, TX, USA) with 1 × 10^5^ cells per sample. After washing, cells were resuspended in MitoPotential assay buffer according to the manufacturer’s instructions and stained with MitoPotential dye for 20 min at 37 °C in the dark. Immediately before acquisition, 7-AAD was added and samples were incubated for 5 min at RT in the dark, followed by analysis on the Guava^®^ Muse™ Cell Analyzer (Luminex Corporation, Austin, TX, USA). The proportion of cells displaying mitochondrial depolarization, as well as the distribution of live, depolarized live, depolarized/dead and dead cells, was determined.

#### 4.6.3. Reactive Oxygen Species (ROS) Analysis

Intracellular ROS levels were quantified using 1 × 10^5^ cells with the Muse™ Oxidative Stress Kit (Luminex Corporation, Austin, TX, USA). After washing with DPBS, cells were resuspended in the Oxidative Stress assay buffer and incubated with the ROS detection reagent for 30 min at 37 °C in the dark. Stained cells were analyzed on the Guava^®^ Muse™ Cell Analyzer (Luminex Corporation, Austin, TX, USA), and results were expressed as the percentages of ROS-negative and ROS-positive cells.

### 4.7. Metabolomic Analysis

#### 4.7.1. Extracellular Metabolites

After 72 h statins exposure, culture medium was collected and centrifuged at 500× *g* for 4 min at 13 °C. Supernatants were transferred into clean tubes and stored at −80 °C until analysis. Fresh complete medium incubated under identical conditions without cells served as background reference. For the ^1^H-NMR analysis, 500 µL of culture medium was mixed with 100 µL of NMR solution (consisting of 200 mM phosphate buffer with pH 7.4 in D_2_O, enriched with [sodium 3-(trimethylsilyl)propionic-2,2,3,3-d_4_] (TMS-d_4_) to a level of 0.2 mM and transferred to an NMR tube. The ^1^H-NMR spectra were recorded on a Bruker Avance III, 600 MHz, equipped with a TCI cryoprobe, and subsequently processed as already described.

#### 4.7.2. Intracellular Metabolites

Cells were detached using TrypLE™ Express (Gibco, Thermo Fisher Scientific, Waltham, MA, USA), washed in DPBS, and counted; 1.5 × 10^6^ cells were collected per sample. Pellets were centrifuged (500× *g* for 4 min at 13 °C) and stored at −80 °C. For extraction, pellets were thawed on ice, mixed with 100 μL ice-cold 100% isopropanol (Sigma–Aldrich, St. Louis, MO, USA), and lysed by three cycles of water-bath ultrasonication for 3 min (ProbeTec, Becton Dickinson), alternating with liquid nitrogen immersion (30 s). Lysates were centrifuged at 30,000× *g* for 5 min at 2 °C, and supernatants were transferred to new tubes for NMR-based metabolomic analysis. The supernatant was dried in a SpeedVac vacuum dryer (ThermoScientific, Waltham, MA, USA). The dry matter was dissolved in 550 µL of phosphate-buffered deuterated water (500 mM, pH 7.4, which contained 0.2 mM TMS-d_4_ (trimethylsilylpropionic acid-d_4_) as a chemical shift reference) and was assigned a chemical shift of 0.000 ppm during data processing. Extraction was performed following a Biocrates-based protocol with minor adjustments [44,45,46]. Metabolite abundances were normalized to cell number.

#### 4.7.3. NMR Measurement

NMR data were acquired using a 600 MHz Avance III NMR spectrometer (Bruker, Munich, Germany) equipped with a cryoprobe at an acquisition temperature of T = 310 K. The samples were freshly prepared and tempered for 5 min at 310 K before measurement. 1D and 2D NMR spectra were measured for each sample. Standard metabolomic profiling protocols from Bruker were modified as follows: (i) NOESY with pre-saturation (noesygppr1d): FID size 64k, dummy scans: 4, number of scans: 128, spectral width: 20.4750 ppm; (ii) cpmg with pre-saturation: FID size 4k, dummy scans: 8, number of scans: 512 for cell media, 4096 for cell lysates, spectral width: 16.0125 ppm; (iii) homonuclear J-resolved spectra: FID size 8k, dummy scans: 16, number of scans: 64, COSY with pre-saturation (cosygpprqf) acquired for two randomly chosen media and two lysates: FID size 4k, dummy scans: 8, number of scans: 32, spectral width 16.0125 ppm. All the experiments were performed with a relaxation delay of 4 s.

#### 4.7.4. Data Analysis

NMR spectra were binned to 0.001 ppm, ranging from 0.00 to 10.00 ppm. The multiplicity of the peaks was confirmed in J-resolved spectra, and the homonuclear cross-peaks were confirmed in COSY spectra. Spectra were solved using an online metabolomics database (www.hmdb.ca, accessed 11-12/2025), a free trial version of Chenomx software (NMR suite 9.0), an in-house metabolite database, and metabolomics literature searches. Subsequently, after identifying the metabolites, subregions of the spectra were selected, to which only one metabolite was assigned or minimally influenced by other co-occurring metabolites. The integrals of the selected metabolites were calculated from the binned spectra (size 0.001 ppm). These data express the relative concentration of a particular metabolite in the sample. Metabolites showing weakly intense peaks or strongly overlapping peaks were excluded from the quantitative evaluation.

### 4.8. Statistical Analysis

MTT analyses were conducted in R (version 4.4.2) using the packages listed in the References section. Exploratory data analysis was first performed, with continuous variables summarized by medians and lower and upper quartiles, and categorical variables by counts and percentages. Normalized absorbance values (viability relative to control) and their percentage deviations were visualized using spaghetti plots to assess variability across time and treatment conditions. Because the dataset included repeated measurements across multiple time points, a linear mixed-effects model was fitted. The response variable was the transformed absorbance value, and the fixed-effects structure included the full interaction of cell line, concentration, time, and statin. A random intercept was included to account for within-replicate dependence. To identify an appropriate transformation of the response, the powerTransform() function from the car package v3.13 was applied. Model diagnostics were performed to evaluate assumptions and optimize model fit. Post hoc analyses were conducted using estimated marginal means for combinations of line and time, comparing each statin treatment with the corresponding control. *p*-values were adjusted for multiple comparisons using Tukey’s method. All marginal means were back-transformed to the original scale for interpretation [47,48,49,50,51].

## 5. Conclusions

This study provides an integrated functional characterization of all clinically available statins in three novel patient-derived PDAC cell lines. Under standardized conditions, lipophilic statins consistently reduced cell viability more effectively than hydrophilic agents and induced coordinated alterations in mitochondrial membrane potential, ROS production, cell-cycle progression, and apoptotic cell death. NMR-based metabolic profiling further showed that statin exposure globally attenuates glycolytic and amino-acid dependent pathways in PDAC cells, while preserving line-specific metabolic fingerprints, particularly in the handling of glucose, lactate, branched-chain amino acids, and the glutamine–glutamate pathway.

Our results reveal pronounced functional heterogeneity among the three patient-derived PDAC cell lines. PDAC-1 exhibited high statin sensitivity, with robust G0/G1 arrest, mitochondrial depolarization, ROS elevation, and Annexin V-positive apoptosis. PDAC-3 showed an intermediate phenotype with substantial cytostatic and pro-apoptotic responses, whereas PDAC-2, characterized by high basal ROS levels, a hyperproliferative cell-cycle profile, and enhanced glycolytic flux, displayed ROS reduction, only modest inhibition of proliferation and relative resistance to apoptosis despite clear mitochondrial dysfunction. These divergent patterns suggest that differences in redox homeostasis, mitochondrial resilience, cell cycle control, and apoptotic competence may contribute to variability in statin associated responses across PDAC cell lines.

Consistent with preclinical studies in other solid tumors, these findings provide a rationale to further evaluate statins in combination settings, where they may act as modulators of tumor cell fitness rather than as standalone cytotoxic agents [52,53,54]. The marked inter-tumoral heterogeneity observed here indicates that individual PDAC tumors may exhibit distinct statin sensitivity profiles. Because the present in vitro system does not capture the influence of the tumor immune and stromal microenvironment, these observations require validation in appropriate in vivo models and more complex experimental systems. Overall, the present results are hypothesis-generating and motivate future in vivo and combination studies to directly test potential chemosensitizing effects and the role of microenvironmental context.

In summary, our results provide a preclinical framework suggesting that integrated functional profiling of patient-derived PDAC cell lines may help stratify tumor subsets for the evaluation of statin-containing combination therapies. Further validation in relevant in vivo PDAC models will be necessary before considering translational applications.

## Figures and Tables

**Table 1:** Integrated functional and metabolic associations underlying differential statin responses in patient-derived PDAC cell lines (**↑** increased activity, **↓** decreased activity).

Result	PDAC-1	PDAC-2	PDAC-3	Association
**Morphological response**	Strong loss of confluence, rounding, detachment	Aggregate formation, stress morphology without massive loss	Moderate disruption, partial detachment	Morphological stress alone does not predict cytotoxicity
**Loss of viability (MTT assay)**	Strong, time- and dose-dependent	Modest, delayed	Intermediate	Functional statin sensitivity differs markedly between lines
**Basal intracellular ROS**	Low–intermediate	High	Low	Basal redox state differs intrinsically
**ROS response to statins**	↑ (especially LO, SI)	↓ (all statins)	↑ (pronounced)	Pro-oxidant shift associates with sensitivity
**Mitochondrial dysfunction (Δψm loss and downstream fate)**	Pronounced Δψm loss efficiently converted to apoptosis	Δψm loss with limited apoptotic conversion	Δψm loss with partial apoptotic conversion	Mitochondrial depolarization is a common upstream effect; cell fate is determined downstream
**Basal cell-cycle profile**	Balanced distribution	Hyperproliferative (S+G2/M-dominant)	G0/G1-enriched	High proliferative drive confers resistance
**Statin-induced cell-cycle arrest**	Pronounced G0/G1 accumulation	Weak cytostatic effect	Pronounced G0/G1 accumulation	Cytostatic response correlates with sensitivity
**Apoptosis (Annexin V+)**	Robust induction	Limited induction	Robust induction	Apoptotic competence is decisive
**Basal glycolytic activity**	Intermediate	High glucose uptake, high lactate release	Low glycolytic flux	Distinct metabolic programs
**Metabolic response to statins**	Global attenuation of glucose and amino-acid utilization	Preserved metabolic activity, high adaptability	Attenuation with line-specific features	Metabolic plasticity supports resistance

**Table 2:** Clinicopathological characteristics of PDAC-derived cell lines.

	PDAC-1	PDAC-2	PDAC-3
Patient sex	female	male	male
Age at diagnosis	49	60	64
Tumor origin	Primary tumor	Primary tumor	Primary tumor
Tumor location	Tail of pancreas	Head of pancreas	Pancreas (total duodenopancreatectomy)
Histological diagnosis	PDAC	PDAC	High-grade PDAC
Histological grade	G3	G2	G3
Pathological stage	pT3 pN1 pMx	pT2 pN2 pMx	pT2 pN2

## Data Availability

The original contributions presented in this study are included in the article/Supplementary Materials. Further inquiries can be directed to the corresponding author. The patient-derived PDAC cell lines generated in this study are available from Jan Strnadel (jan.strnadel@uniba.sk) upon reasonable request. The raw and evaluated NMR data are available upon reasonable request from: eva.baranovicova@uniba.sk.

## Supplementary Materials

The following supporting information can be downloaded at: https://www.mdpi.com/article/10.3390/ijms27072972/s1. References [48,49,50,51,55,56,57,58,59,60,61,62] are cited in the supplementary materials.

## Abbreviations

The following abbreviations are used in this manuscript:

AT	Atorvastatin
CTRL	Control
DMEM	Dulbecco’s Modified Eagle Medium
DPBS	Dulbecco’s Phosphate-Buffered Saline
FBS	Fetal bovine serum
FL	Fluvastatin
HMG-CoA	3-hydroxy-3-methylglutaryl-coenzyme A
LO	Lovastatin
MTT	3-(4,5-dimethylthiazol-2-yl)-2,5-diphenyltetrazolium bromide
PDAC	Pancreatic ductal adenocarcinoma
PI	Pitavastatin
PR	Pravastatin
RO	Rosuvastatin
ROS	Reactive oxygen species
RT	Room temperature
SDS	Sodium dodecyl sulfate
SI	Simvastatin
