# Supplementary material for: Anti-Tumor Effects of Statins in Pancreatic Ductal Adenocarcinoma Cells
Source: Int J Mol Sci. 2026 Mar 25;27(7):2972. doi: 10.3390/ijms27072972 (PMC13073267; doi:10.3390/ijms27072972)
Supplement: Supplementary file 1 [file ijms-27-02972-s001.zip › ijms-4175050-supplementary.pdf]

International Journal of *Molecular Sciences*

MDPI

Supplementary material

Anti-Tumor Effects of Statins in Pancreatic Ductal Adenocarcinoma Cells

Veronika Kucháriková [1,2], Zuzana Hatoková [1], Eva Baranovičová [1], Bibiána Baďurová [1], Tereza Pavlišová [1], Lucia Kotúľová [1], Michal Kalman [3], Juraj Marcinek [3], Oľga Chodelková [1,2], Slavomíra Nováková [1], Ján Strnádel [1], Henrieta Škovierová [1,*] and Erika Halašová [1]

[1] Biomedical Centre Martin, Jessenius Faculty of Medicine in Martin, Comenius University in Bratislava, Malá Hora 4C, 036 01 Martin, Slovakia; kucharikova14@uniba.sk (V.K.); zuzana.hatokova@uniba.sk (Z.H.); eva.baranovicova@uniba.sk (E.B.); bibiana.badurova@uniba.sk (B.B.); tereza.pavlisova@uniba.sk (T.P.); lucia.kotulova@uniba.sk (L.K.); ticha24@uniba.sk (O.C.); slavomira.novakova@uniba.sk (S.N.); jan.strnadel@uniba.sk (J.S.); erika.halasova@uniba.sk (E.H.)

[2] Department of Medical Biochemistry, Jessenius Faculty of Medicine in Martin, Comenius University in Bratislava, Malá Hora 4D, 036 01 Martin, Slovakia

[3] Department of Pathological Anatomy, Jessenius Faculty of Medicine in Martin, Comenius University in Bratislava and University Hospital Martin, Kollárová 2, 036 01 Martin, Slovakia; michal.kalman@uniba.sk (M.K.); juraj.marcinek@uniba.sk (J.M.)

* Correspondence: henrieta.skovierova@uniba.sk; Tel.: +421-432-633-904

Academic Editor: Firstname Lastname

Received: date
Revised: date
Accepted: date
Published: date

Citation: To be added by editorial staff during production.

Copyright: © 2025 by the authors. Submitted for possible open access publication under the terms and conditions of the Creative Commons Attribution (CC BY) license (https://creativecommons.org/licenses/by/4.0/).

The R programming language (ver. 4.4.2) was used together with libraries listed in the References section. Exploratory data analysis (EDA) summarized the percentage of cells using the median (lower–upper quartile) and visualized distributions across groups using boxplots. To address the study hypotheses on differences between statin-treated groups and the control (CTRL), robust linear regression models were fitted with Statin and Status/Phase as predictors, including their interaction where relevant. The hypotheses were then evaluated using planned post hoc contrasts of estimated marginal means (emmeans), comparing each statin to CTRL within the corresponding Status/Phase strata, with p-values adjusted for multiple testing using Dunnett's method [1-13].

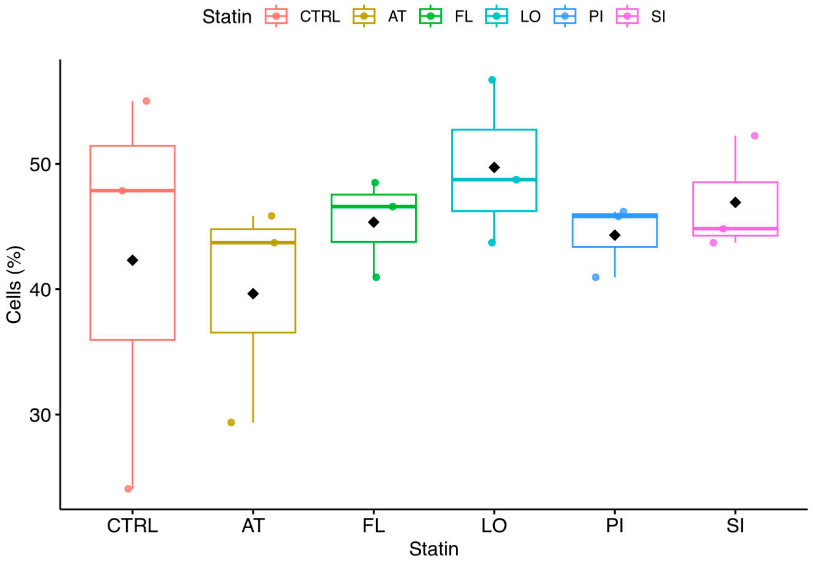

Figure S1. Percentage of ROS-positive cells by statin group. Boxplots display the percentage of ROS-positive cells for each statin group: line = median, box = lower/upper quartile, whiskers = most extreme values within 1.5×(upper–lower quartile), points beyond

whiskers = potential outliers, black diamond = mean. Individual points represent values from the individual PDAC cell lines (*n* = 3).

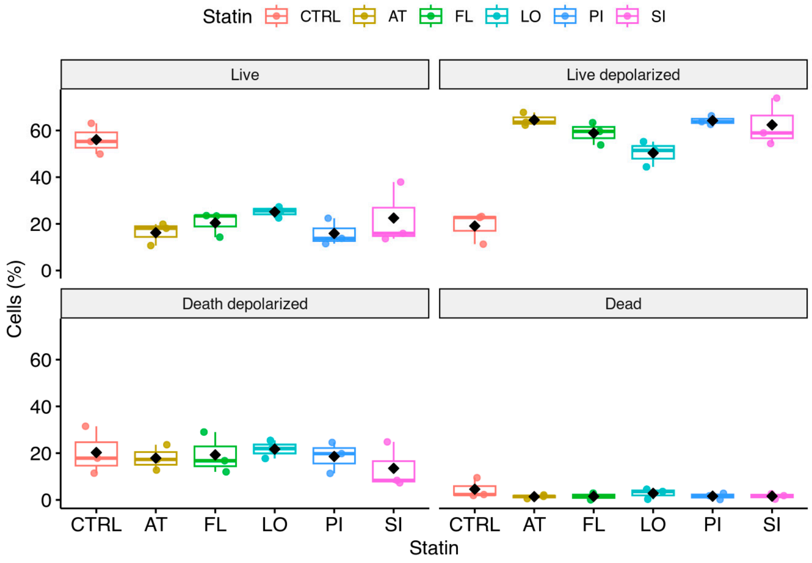

Figure S2. Mitochondrial membrane potential categories by statin group. Boxplots show the percentage of cells in each mitochondrial status category (Live, Live depolarized, Death depolarized, Dead) for each statin group. Boxplots display Cells (%) by statin group: line = median, box = lower/upper quartile, whiskers = most extreme values within 1.5×(upper–lower quartile), points beyond whiskers = potential outliers, black diamond = mean. Individual points represent values from the individual PDAC cell lines (*n* = 3).

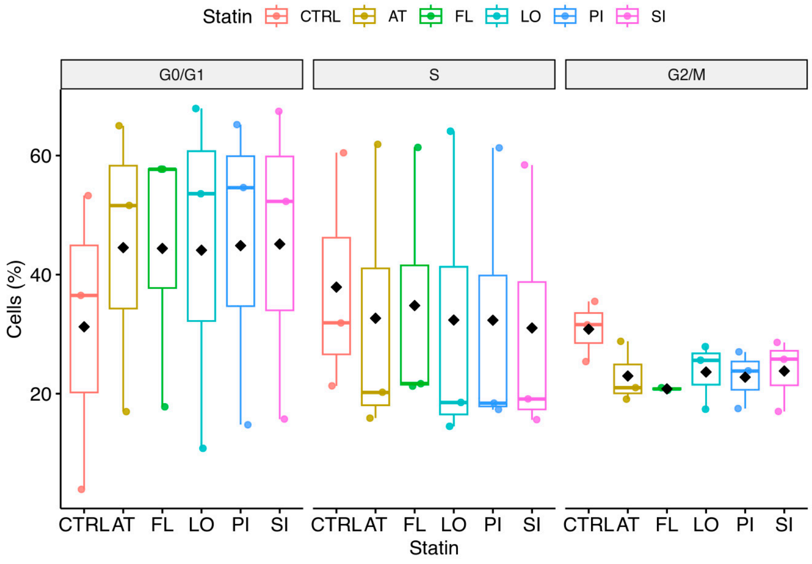

Figure S3. Cell-cycle phase distribution by statin group. Boxplots show the percentage of cells in each cell-cycle phase (G0/G1, S, G2/M) for each statin group. Boxplots display Cells (%) by statin group: line = median, box = lower/upper quartile, whiskers = most extreme values within 1.5×(upper–lower quartile), points beyond whiskers = potential outliers, black diamond = mean. Individual points represent values from the individual PDAC cell lines (*n* = 3).

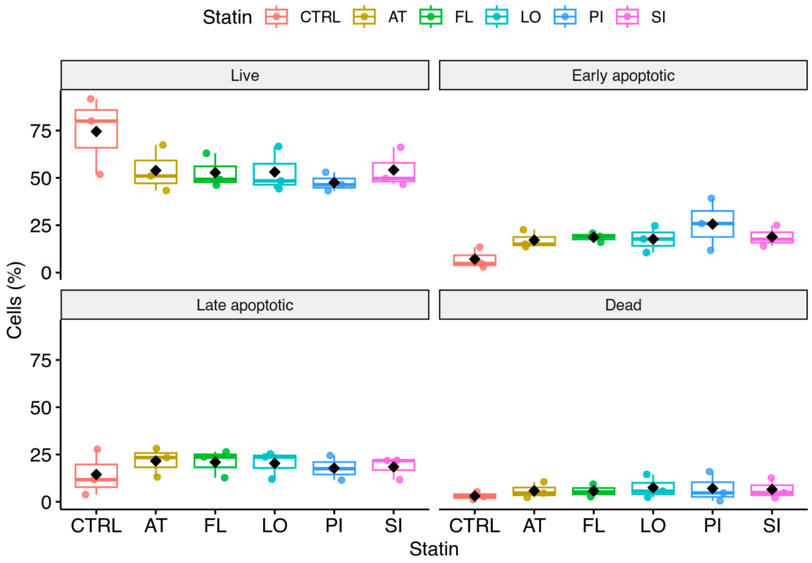

Figure S4. Annexin V/7-AAD apoptosis categories by statin group. Boxplots show the percentage of cells classified as Live, Early apoptotic, Late apoptotic, or Dead based on Annexin V/7-AAD staining for each statin group. Boxplots display Cells (%) by statin group: line = median, box = lower/upper quartile, whiskers = most extreme values within 1.5×(upper–lower quartile), points beyond whiskers = potential outliers, black diamond = mean. Individual points represent values from the individual PDAC cell lines (n = 3).

PDAC-1

significance

5 µM	24h	48h	72h	96h	120h
AT	ns	***	***	***	***
FL	ns	ns	***	***	***
LO	ns	ns	***	***	***
PI	ns	*	***	***	***
PR	ns	ns	**	ns	ns
RO	ns	**	**	***	*
SI	ns	*	***	***	***

10 µM	24h	48h	72h	96h	120h
AT	ns	***	***	***	***
FL	ns	***	***	***	***
LO	ns	***	***	***	***
PI	ns	***	***	***	***
PR	ns	ns	ns	***	ns
RO	ns	***	***	***	***
SI	ns	***	***	***	***

20 µM	24h	48h	72h	96h	120h
AT	ns	***	***	***	***
FL	ns	***	***	***	***
LO	ns	***	***	***	***
PI	ns	***	***	***	***
PR	ns	**	ns	ns	ns
RO	ns	***	***	***	***
SI	ns	***	***	***	***

p-value

5 µM	24h	48h	72h	96h	120h
AT	0.9923	3.9e-04	< 1e-16	< 1e-16	< 1e-16
FL	0.5433	0.1022	< 1e-16	< 1e-16	< 1e-16
LO	0.3159	0.4965	4.4e-16	< 1e-16	< 1e-16
PI	0.9874	0.0424	< 1e-16	< 1e-16	< 1e-16
PR	0.2494	0.0527	0.0030	0.1004	0.7697
RO	9.53e-01	1.3e-03	1.7e-03	3.6e-05	1.06e-02
SI	0.7265	0.0171	< 1e-16	< 1e-16	< 1e-16

10 µM	24h	48h	72h	96h	120h
AT	0.9999	2.6e-06	< 1e-16	< 1e-16	< 1e-16
FL	0.2950	4.0e-14	< 1e-16	< 1e-16	< 1e-16
LO	0.3742	3.3e-13	< 1e-16	< 1e-16	< 1e-16
PI	0.9041	9.2e-12	< 1e-16	< 1e-16	< 1e-16
PR	0.6700	0.1167	0.4886	2.3e-04	0.4432
RO	0.5508	3.5e-06	9.5e-15	< 1e-16	< 1e-16
SI	0.1409	< 1e-16	< 1e-16	< 1e-16	< 1e-16

20 µM	24h	48h	72h	96h	120h
AT	0.9919	4.6e-07	<1e-16	< 1e-16	<1e-16
FL	0.9726	1.0e-06	<1e-16	< 1e-16	<1e-16
LO	0.9999	4.3e-10	<1e-16	< 1e-16	<1e-16
PI	0.9995	1.5e-07	<1e-16	< 1e-16	<1e-16
PR	0.8330	0.0037	0.8988	0.6248	0.9990
RO	0.9683	5.4e-06	4.0e-11	< 1e-16	<1e-16
SI	0.9950	4.8e-05	<1e-16	< 1e-16	<1e-16

PDAC-2

significance

5 µM	24h	48h	72h	96h	120h
AT	ns	ns	ns	ns	ns
FL	ns	*	ns	***	*
LO	ns	ns	ns	ns	ns
PI	ns	ns	ns	*	**
PR	ns	ns	ns	ns	ns
RO	ns	ns	ns	***	***
SI	ns	ns	ns	ns	ns

10 µM	24h	48h	72h	96h	120h
AT	ns	ns	ns	***	***
FL	ns	ns	ns	***	***
LO	ns	ns	ns	ns	***
PI	ns	ns	ns	***	***
PR	***	***	ns	ns	ns
RO	***	**	ns	***	***
SI	***	ns	ns	ns	*

20 µM	24h	48h	72h	96h	120h
AT	ns	ns	ns	***	***
FL	ns	ns	ns	**	***
LO	ns	ns	ns	ns	**
PI	ns	ns	ns	***	***
PR	**	ns	ns	ns	ns
RO	ns	***	**	***	***
SI	***	ns	ns	*	***

p-value

5 µM	24h	48h	72h	96h	120h
AT	0.9994	0.9877	0.9936	0.4048	0.2462
FL	0.9605	0.0171	0.4299	6.4e-05	0.0311
LO	0.5604	0.8097	0.9448	0.0593	0.9672
PI	0.8993	0.9405	0.9999	0.0173	0.0020
PR	0.1453	0.7695	0.9976	0.8579	0.7576
RO	0.2812	0.9941	0.3220	1.0e-06	9.7e-08
SI	0.4833	0.4877	0.8960	0.9661	0.8275

10 µM	24h	48h	72h	96h	120h
AT	0.9796	0.0865	0.8680	1.1e-10	< 1e-16
FL	0.7623	0.8683	0.9999	8.9e-08	< 1e-16
LO	0.9998	0.9910	0.0867	0.2651	7.8e-06
PI	0.7504	0.9996	0.9774	1.3e-11	< 1e-16
PR	6.5e-14	1.9e-07	0.9955	0.8314	0.1814
RO	2.7e-07	0.0029	0.5335	3.3e-05	2.7e-08
SI	3.3e-05	0.6654	0.7700	0.9957	0.0141

20 µM	24h	48h	72h	96h	120h
AT	0.9961	0.1981	0.3063	3.1e-06	< 1e-16
FL	0.9984	0.9724	0.8013	0.0018	< 1e-16
LO	0.9837	0.9470	0.9197	0.9979	0.0087
PI	0.5783	0.9997	0.9901	4.3e-06	< 1e-16
PR	0.0055	0.4364	0.9261	0.8575	0.3505
RO	0.9421	6.3e-04	0.0047	5.1e-05	2.6e-09
SI	6.8e-05	0.9785	0.6627	0.0374	5.9e-08

PDAC-3

significance

5 µM	24h	48h	72h	96h	120h
AT	ns	ns	***	***	ns
FL	ns	ns	***	***	***
LO	ns	ns	***	***	ns
PI	ns	ns	***	***	***
PR	ns	ns	ns	**	***
RO	ns	ns	ns	***	***
SI	ns	ns	**	ns	ns

10 µM	24h	48h	72h	96h	120h
AT	ns	ns	***	***	***
FL	ns	**	***	***	***
LO	ns	ns	***	***	***
PI	ns	**	***	***	***
PR	ns	ns	ns	ns	***
RO	ns	*	*	ns	***
SI	ns	ns	***	***	***

20 µM	24h	48h	72h	96h	120h
AT	ns	ns	***	***	***
FL	ns	*	***	***	***
LO	ns	***	***	***	***
PI	ns	***	***	***	***
PR	ns	*	ns	***	***
RO	ns	***	ns	ns	**
SI	ns	***	***	***	***

p-value

5 µM	24h	48h	72h	96h	120h
AT	0.7166	0.9847	1.2e-09	4.1e-08	0.9999
FL	0.6413	0.8973	9.5e-15	< 1e-16	1.3e-06
LO	0.8785	0.9999	1.2e-05	9.1e-04	0.0795
PI	0.9044	0.2784	< 1e-16	< 1e-16	< 1e-16
PR	0.9998	0.9717	0.2212	0.0017	2.8e-08
RO	0.9987	0.9460	0.9785	7.6e-04	< 1e-16
SI	0.6190	0.9339	0.0011	0.0782	0.2232

10 µM	24h	48h	72h	96h	120h
AT	0.3972	0.6069	2.2e-16	< 1e-16	< 1e-16
FL	0.1819	0.0024	< 1e-16	< 1e-16	< 1e-16
LO	0.2671	0.8298	6.5e-07	1.6e-11	4.3e-05
PI	0.2676	0.0025	< 1e-16	< 1e-16	< 1e-16
PR	0.8443	0.8616	0.2989	0.1407	4.6e-05
RO	0.0544	0.0412	0.0116	0.9932	5.1e-11
SI	0.1374	0.8202	2.1e-07	1.1e-13	4.6e-11

20 µM	24h	48h	72h	96h	120h
AT	0.9925	0.1415	< 1e-16	< 1e-16	< 1e-16
FL	0.8846	0.0131	< 1e-16	< 1e-16	< 1e-16
LO	0.9114	8.2e-09	6.7e-14	< 1e-16	< 1e-16
PI	0.9999	8.1e-09	< 1e-16	< 1e-16	< 1e-16
PR	0.9994	0.0452	0.0518	7.2e-05	4.8e-12
RO	0.9999	3.9e-05	0.6829	0.2270	0.0055
SI	0.8653	2.0e-15	2.2e-16	< 1e-16	< 1e-16

Figure S5. Full statistical output for the MTT viability test. Tables summarize the statistical comparisons of each statin treatment versus the corresponding DMSO control within each PDAC cell line (PDAC-1, PDAC-2, PDAC-3) at each concentration (5, 10, 20 µM) and time point (24–120 h) (n = 3). For each setting, the left table reports significance codes (***p<0.001,

https://doi.org/10.3390/xxxxx

**$p<0.01$, *$p<0.05$, ns – not significant) and the corresponding numerical p-values are provided in separate tables (right) in scientific notation. Abbreviations: atorvastatin (AT), fluvastatin (FL), lovastatin (LO), pitavastatin (PI), pravastatin (PR), rosuvastatin (RO), simvastatin (SI).

References

1. R Core Team. R: A Language and Environment for Statistical Computing; R Foundation for Statistical Computing: Vienna, Austria, 2024. Available online: https://www.r-project.org/ (accessed on 19 March 2026).
2. Fox, J.; Weisberg, S.; Price, B. car: Companion to Applied Regression, Version 3.1-3. 2024. Available online: https://cran.r-project.org/web/packages/car/car.pdf (accessed on 19 March 2026).
3. Wickham, H.; François, R.; Henry, L.; Müller, K.; Vaughan, D. *dplyr*: A Grammar of Data Manipulation, Version 1.1.4. 2023. Available online: https://cran.r-project.org/web/packages/dplyr/dplyr.pdf (accessed on 19 March 2026).
4. Lenth, R.V. *emmeans*: Estimated Marginal Means, aka Least-Squares Means, Version 1.11.1. 2025. Available online: https://cran.r-project.org/web/packages/emmeans/emmeans.pdf (accessed on 19 March 2026).
5. Gohel, D.; Skintzos, P. *flextable*: Functions for Tabular Reporting, Version 0.9.8. 2025. Available online: https://cran.r-project.org/web/packages/flextable/flextable.pdf (accessed on 19 March 2026).
6. Wickham, H.; Chang, W.; Henry, L.; Pedersen, T.L.; Takahashi, K.; Wilke, C.; Woo, K.; Yutani, H.; Dunnington, D.; van den Brand, T. *ggplot2*: Create Elegant Data Visualisations Using the Grammar of Graphics, Version 3.5.2. 2025. Available online: https://cran.r-project.org/web/packages/ggplot2/ggplot2.pdf (accessed on 19 March 2026).
7. Kassambara, A. *ggpubr*: 'ggplot2' Based Publication Ready Plots, Version 0.6.0. 2023. Available online: https://cran.r-project.org/web/packages/ggpubr/ggpubr.pdf (accessed on 19 March 2026).
8. Simpson, G.L. *gratia*: Graceful 'ggplot'-Based Graphics and Other Functions for GAMs Fitted Using 'mgcv', Version 0.10.0. 2024. Available online: https://cran.r-project.org/web/packages/gratia/gratia.pdf (accessed on 19 March 2026).
9. Sjoberg, D.D.; Larmarange, J.; Curry, M.; de la Rua, E.; Lavery, J.; Whiting, K.; et al. *gtsummary*: Presentation-Ready Data Summary and Analytic Result Tables, Version 2.5.0. 2025. Available online: https://cran.r-project.org/web/packages/gtsummary/gtsummary.pdf (accessed on 19 March 2026).
10. Lüdecke, D. *sjPlot*: Data Visualization for Statistics in Social Science, Version 2.8.17. 2024. Available online: https://cran.r-project.org/web/packages/sjPlot/sjPlot.pdf (accessed on 19 March 2026).
11. Wickham, H.; Vaughan, D.; Girlich, M. *tidyr*: Tidy Messy Data, Version 1.3.1. 2024. Available online: https://cran.r-project.org/web/packages/tidyr/tidyr.pdf (accessed on 19 March 2026).
12. Sarkar, D. *lattice*: Trellis Graphics for R, version 0.22-7; 2025. Available online: https://cran.r-project.org/web/packages/lattice/lattice.pdf (accessed on 19 March 2026).
13. Bates, D.; Maechler, M.; Bolker, B.; Walker, S. *lme4*: Linear Mixed-Effects Models Using 'Eigen' and S4, Version 1.1-37. 2025. Available online: https://cran.r-project.org/web/packages/lme4/lme4.pdf (accessed on 19 March 2026).

https://doi.org/10.3390/xxxxx
